# Cyclic Tensile Strain Can Play a Role in Directing both Intramembranous and Endochondral Ossification of Mesenchymal Stem Cells

**DOI:** 10.3389/fbioe.2017.00073

**Published:** 2017-11-27

**Authors:** Simon F. Carroll, Conor T. Buckley, Daniel J. Kelly

**Affiliations:** ^1^Trinity Centre for Bioengineering, Trinity Biomedical Sciences Institute, Trinity College Dublin, Dublin, Ireland; ^2^Department of Mechanical and Manufacturing Engineering, School of Engineering, Trinity College Dublin, Dublin, Ireland; ^3^Department of Anatomy, Royal College of Surgeons in Ireland, Dublin, Ireland; ^4^Advanced Materials and Bioengineering Research Centre (AMBER), Royal College of Surgeons in Ireland and Trinity College Dublin, Dublin, Ireland

**Keywords:** mesenchymal stem cells, endochondral ossification, intramembranous ossification, tensile strain, mechanical stimulation, osteogenesis, chondrogenesis

## Abstract

Successfully regenerating damaged or diseased bone and other joint tissues will require a detailed understanding of how joint specific environmental cues regulate the fate of progenitor cells that are recruited or delivered to the site of injury. The goal of this study was to explore the role of cyclic tensile strain (CTS) in regulating the initiation of mesenchymal stem cell/multipotent stromal cell (MSC) differentiation, and specifically their progression along the endochondral pathway. To this end, we first explored the influence of CTS on the differentiation of MSCs in the absence of any specific growth factor, and secondly, we examined the influence of the long-term application of this mechanical stimulus on markers of endochondral ossification in MSCs maintained in chondrogenic culture conditions. A custom bioreactor was developed to apply uniaxial tensile deformation to bone marrow-derived MSCs encapsulated within physiological relevant 3D fibrin hydrogels. Mechanical loading, applied in the absence of soluble differentiation factors, was found to enhance the expression of both tenogenic (COL1A1) and osteogenic markers (BMP2, RUNX2, and ALPL), while suppressing markers of adipogenesis. No evidence of chondrogenesis was observed, suggesting that CTS can play a role in initiating direct intramembranous ossification. During long-term culture in the presence of a chondrogenic growth factor, CTS was shown to induce MSC re-organization and alignment, increase proteoglycan and collagen production, and to enhance the expression of markers associated with endochondral ossification (BMP2, RUNX2, ALPL, OPN, and COL10A1) in a strain magnitude-dependent manner. Taken together, these findings indicate that tensile loading may play a key role in promoting both intramembranous and endochondral ossification of MSCs in a context-dependent manner. In both cases, this loading-induced promotion of osteogenesis was correlated with an increase in the expression of the osteogenic growth factor BMP2. The results of this study demonstrate the potent role that extrinsic mechanical loading plays in guiding stem cell fate, which must be carefully considered when designing cell and tissue-engineering therapies if they are to realize their clinical potential.

## Introduction

Understanding the etiology of diseases such as osteoarthritis requires an in-depth understanding of the role that joint specific environmental factors play in the maintenance of cartilage and its conversion into bone. Furthermore, successfully regenerating damaged or diseased bone and other joint tissues also requires an understanding of how such environmental factors regulate the fate of cells that are recruited or delivered to the site of injury. Multipotent stromal cells (MSCs) isolated from bone marrow can be induced to differentiate toward multiple lineages, making them an attractive cell type for cartilage and bone tissue engineering (Caplan, 1991; Yoo et al., 1998; Steinert et al., 2012; Vinardell et al., 2012; Grayson et al., 2015). Bone development occurs *via* two distinct mechanisms—intramembranous or endochondral ossification. Intramembranous ossification involves the direct differentiation of MSCs into osteoblasts, whereas endochondral ossification first involves differentiation of precursor cells into chondrocytes, the formation of a cartilage template and its subsequent replacement by bone (Frohlich et al., 2008). The latter is the process by which all long bones are formed. It has long been suggested that endochondral ossification during limb development is mechanoregulated, with early studies linking femoral ossification to the regions of maximal tensile strains (Carey, 1922). More recent work has confirmed this relationship between the mechanical environment and bone development (Nowlan et al., 2010). It has also been reported that, during bone healing, callus distraction can produce a systemic increase in concentrations of several bone growth factors (Sato et al., 1999; Weiss et al., 2002). While clearly mechanical cues are integral to directing bone growth, development, and healing, the underlying mechanisms remain poorly understood. A more complete understanding of these processes will benefit not only the fields of cartilage and bone tissue engineering but will also be integral to the development of novel therapeutic strategies to treat complex diseases such as osteoarthritis.

There has been increased interest in the use of *in vitro* bioreactor systems to examine how different mechanical stimuli regulate both osteogenesis and chondrogenesis of stem cells (Kelly and Jacobs, 2010). It has been demonstrated that the application of uniaxial cyclic tensile strain (CTS) to MSCs cultured on 2D substrates can induce osteogenic and fibrogenic gene expression (Simmons et al., 2003; Friedl et al., 2007; Qi et al., 2008, Diederichs et al., 2009). The magnitude of tensile loading has also been shown to be important, with low strains inducing osteogenesis and higher strains promoting the expression of fibrous or myogenic markers (Chen et al., 2008, Jang et al., 2011). These 2D culture systems are generally not considered truly representative of the physiological environment of cells *in vivo*, and MSCs have been shown to respond differentially to tensile loading when seeded on 2D substrates compared with when they are encapsulated within 3D matrices (Rathbone et al., 2012). Of the studies that have explored the influence of tensile strain on directing the differentiation of MSCs in 3D matrices, many have concluded that the application of uniaxial CTS promotes a more ligamentous or fibro-chondrogenic phenotype in MSCs (Altman et al., 2002; Juncosa-Melvin et al., 2007; Farng et al., 2008; Kuo and Tuan, 2008; Connelly et al., 2010; Doroski et al., 2010; Baker et al., 2011; Kreja et al., 2012; Yang et al., 2012). However, it has also been demonstrated that the application of CTS to MSCs in 3D collagen gels in the absence of osteogenic supplements can lead to the upregulation of *BMP2* (Sumanasinghe et al., 2006), suggesting that this mechanical stimulus may also be osteoinductive. Furthermore, it has been reported that tensile strain and dynamic compression differentially regulate MSC differentiation (Haudenschild et al., 2009), with CTS upregulating osteogenic markers *BMP1, ALP*, and *NELL1*, whereas compression was found to promote chondrogenesis of MSCs encapsulated in 3D alginate matrices. This latter finding agrees with a number of separate studies demonstrating that compressive loading can enhance chondrogenesis of MSCs (Huang et al., 2004; Campbell et al., 2006; Thorpe et al., 2012; Steward et al., 2014; Luo et al., 2015). Additionally, it has been demonstrated that high magnitudes of cyclic hydrostatic pressure and dynamic compression can suppress hypertrophy and endochondral ossification of MSCs (Bian et al., 2012; Thorpe et al., 2013; Carroll et al., 2014; Luo et al., 2015, Zhang et al., 2015). Collectively, these studies point to the integral role that mechanical cues play in directing both the initial phenotype of MSCs and in determining their ultimate fate.

Despite the fact that long bones form and regenerate through the process of endochondral ossification, relatively little is known about the role mechanical cues can play in directing this process. Motivated by previous work pointing to a role for tensile strain in directing bone development and healing, the objective of this study was to explore the role of CTS in directing osteogenesis of MSCs within physiologically relevant 3D hydrogels using a custom developed bioreactor system. Recognizing that bone can form and heal through either an intramembranous or an endochondral pathway, we first sought to explore the influence of CTS on the differentiation of MSCs in the absence of specific growth factors to determine if loading alone could initiate osteogenesis. We then examined the influence of the long-term application of this mechanical stimulus on markers of endochondral ossification in MSCs maintained in chondrogenic culture conditions. Our hypothesis was that CTS would enhance endochondral ossification in chondrogenically primed MSCs. The dependency of this process on the magnitude and frequency of the applied strain was also examined.

## Materials and Methods

### CTS Bioreactor

**Figure 1:**
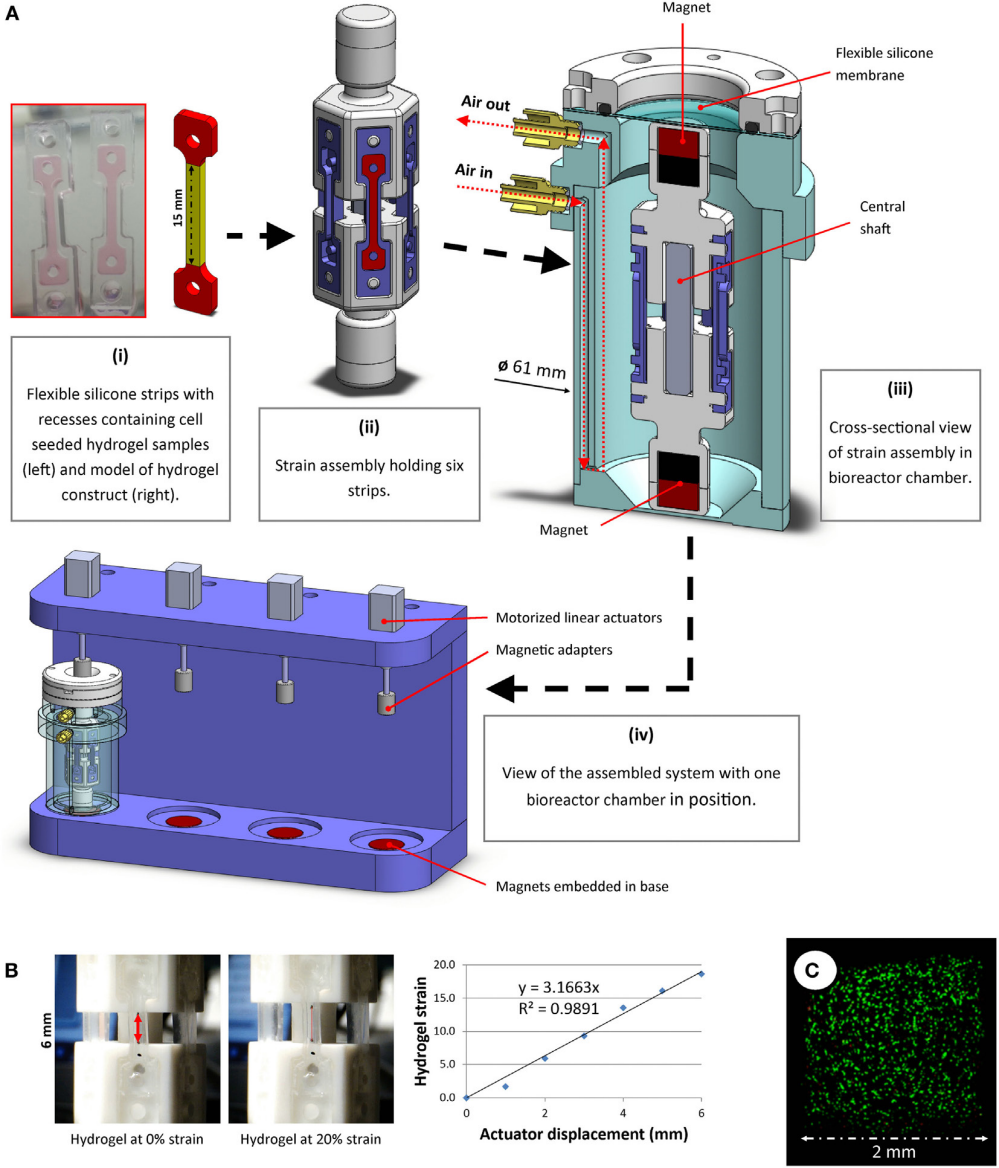
**(A)** A novel bioreactor system for application of uniaxial cyclic tensile strain to soft 3D hydrogels was developed. Dog-bone-shaped cell-seeded hydrogel constructs were molded in recesses within flexible silicone strips. The dimensions of the hydrogel “strain section” (highlighted in yellow) were 15 mm × 2 mm × 1.5 mm (i). These strips were mounted on strain assemblies (ii), which were then sealed into the bioreactor chamber (iii) under sterile conditions. The displacement of external actuators (iv) was aseptically transferred to accurately stretch the strips in the bioreactor using magnets, both outside of and within the chamber, connected around a flexible membrane separator. **(B)** Tensile testing image analyses were performed to correlate actuator displacement with hydrogel strain. Representative images and data from hydrogel strain validation image analyses are shown. **(C)** Multipotent stromal cells encapsulated in hydrogels and cultured in the bioreactor remained viable over 7 days.

A novel bioreactor system was developed for the aseptic application of uniaxial CTS to soft 3D hydrogels. The entire system was housed within a cell culture incubator for the duration of each study. Custom rubber strips (Figure 1A) were molded using a commercially available silicone elastomer kit (Sylgard 184, Dow Corning) by mixing the base with the curing agent at a ratio of 20:1, pouring into a PTFE mold, de-gassing by vacuum and cross-linking for 1 h at 100°C. Each strip had a dog-bone shaped recess (1.5 mm deep) to accommodate hydrogel samples. Gelation of hydrogel samples occurred within these recesses. The strips were soaked and washed in ethanol and water to ensure removal of residual curing agent, then autoclaved before use.

Polymer hexagonal strain mounts were fabricated and assembled. Each strain assembly was designed to allow fixation of six rubber strips, positioned vertically (Figure 1A, ii). Strain assembly parts were free to move along a square shaft, which allowed uniaxial translation whilst constraining rotational movement about the shaft axis. Magnets were embedded at each end of the strain assembly. A cylindrical bioreactor chamber (Figure 1A, iii) was developed to house the strain assembly and maintain samples under sterile conditions. Luer adapters were fitted to air inlet/outlet ports and syringe pumps were used to supply filtered air recycled from within the incubator at a rate of 3 mL/min. When used in conjunction with an oxygen tension controllable incubator, the pumps allowed for gentle agitation and re-oxygenation of the culture medium, thus maintaining uniform dissolved oxygen and nutrient levels at the surface of the samples. A flexible silicone membrane allowed the magnetic adapters outside the chamber to connect to and displace the magnets inside the chamber without compromising the sterility of the chamber. These adapters were fixed to motorized linear actuators (NA08A30, Zaber Technologies Inc., microstep size <0.1 μm). The actuators were mounted on a rig (Figure 1A, iv), into which the bioreactor chambers could be placed. Strong magnets (>20 kg pull force) embedded in the base of this rig below each bioreactor held the lower magnets inside the chamber fixed in position, whereas the magnets attached to the actuators above the bioreactor allowed vertical displacement of the upper magnets within the chamber. Bioreactor components were sterilized using ethylene oxide gas treatment before use.

A custom Matlab (version R2015b, Mathworks Inc.) script was developed that allowed precise control of applied strain, frequency and loading duration. The system was calibrated by mounting hydrogels marked with ink at the extremities and displacing the actuators in 1 mm increments. Image analyses of hydrogels being strained were performed to correlate hydrogel deformation with actuator displacement (Figure 1B). Oxygen levels in the media were monitored near the chamber base using non-invasive oxygen sensors (Fibox 3, PreSens GmbH, data not shown), and MSC viability was monitored and confirmed over the duration of a 7-day pilot study (Figure 1C).

### Cell Isolation and Expansion

Animals were bred and raised for food and not research purposes and were not subject to any scientific procedures before their sacrifice, hence no specific ethical approval was required for this study. Bone marrow-derived MSCs were isolated aseptically from the femoral diaphysis of a single porcine donor for use in multiple studies and expanded *in vitro*, as described previously (Thorpe et al., 2013; Carroll et al., 2014). Briefly, isolated MSCs were plated and cultured in a standard expansion media formulation consisting of high glucose Dulbecco’s Modified Eagle Medium (DMEM, GlutaMAX™) containing 10% fetal bovine serum and 1% penicillin (100 U/mL)–streptomycin (100 mg/mL) (all from GIBCO, Biosciences, Dublin, Ireland) and supplemented with fibroblast growth factor-2 (5 ng/mL; ProSpec-Tany TechnoGene Ltd., Israel) and amphotericin B (0.25 mg/mL; Sigma-Aldrich, Arklow, Ireland). Following colony formation, the cells were trypsinized, counted and plated at a seeding density of 5 × 10^3^ cells/cm^2^ at each passage. Complete media exchanges were performed twice weekly.

### Fibrin Hydrogel Fabrication and Culture

Multipotent stromal cells were suspended in 10,000 KIU/mL aprotinin solution (Nordic Pharma) containing 19 mg/mL sodium chloride and 100 mg/mL bovine fibrinogen type I-S (60–85% protein, 10% sodium citrate, and 15% sodium chloride; Sigma-Aldrich), which was combined 1:1 with 5 U/mL thrombin in 40 mM CaCl_2_ (pH 7) and transferred by syringe to recesses in custom designed flexible silicone strips. These strips were then incubated at 37°C for 45 min to allow cross-linking to complete, producing dog-boned shaped constructs with final concentrations of 50 mg/mL fibrinogen, 2.5 U/mL thrombin, 5,000 KIU/mL aprotinin, 17 mg/mL sodium chloride, 20 mM CaCl_2_, and 10 × 10^6^ cells/mL. The constructs were then loaded into bioreactors containing differentiation medium (DM), consisting of DMEM supplemented with 100 U/mL penicillin/streptomycin (both from Gibco), 100 mg/mL sodium pyruvate, 40 mg/mL l-proline, 50 mg/mL l-ascorbic acid-2-phosphate, 1.5 mg/mL bovine serum albumin, 1× insulin–transferrin–selenium, and 100 nM dexamethasone (all Sigma-Aldrich). For studies requiring chondro-inductive stimuli, constructs were culture in chondrogenic medium [DM supplemented with 10 ng/mL of transforming growth factor-β3 (TGF-β3, ProSpec-Tany TechnoGene, Ltd., Israel)]. Media changes were performed twice weekly.

### Mechanical Stimulation of Hydrogel Encapsulated MSCs

Uniaxial CTS was applied to MSC seeded hydrogels in the bioreactor system described earlier. All bioreactors were housed in incubators set at 5% oxygen, 37°C, and a reciprocating syringe air pump system was employed to pass air bubbles through the culture medium, thereby gently agitating the system and maintaining a uniform dissolved oxygen content of 5%. In the initial study undertaken using differentiation media, hydrogels were deformed at a strain magnitude of 10% and frequency of 0.5 Hz for 4 h each day for 7 days. In the second, long-term study exploring the role of mechanical cues on endochondral ossification, the influence of strain magnitude and frequency was also investigated. Therefore, this study involved four experimental groups (non-loaded control, 5% strain at 0.5 Hz, 10% strain at 0.5 Hz, and 10% strain at 1 Hz) cultured in chondrogenic medium (+TGF-β3) for 21 days.

### Biochemical Analysis

The central (strained) sections of dog-bone constructs (*n* ≥ 3) were used for quantitative analysis of DNA, sulfated glycosaminoglycan (sGAG) and collagen content. The wet weight of each section was measured, then the samples were digested with papain (125 µg/mL) in 0.1 M sodium acetate, 5 mM l-cysteine–HCl, and 0.05 M EDTA (pH 6.0, all Sigma-Aldrich) at 60°C under constant rotation for 18 h. DNA content was quantified using the Hoechst Bisbenzimide 33258 dye assay as described previously (Kim et al., 1988), with a calf thymus DNA standard. Sulfated glycosaminoglycan (GAG) content was quantified using the dimethylmethylene blue dye-binding assay (DMMB; Blyscan, Biocolor Ltd., Northern Ireland) with a chondroitin sulfate standard. Collagen content was determined by measuring the hydroxyproline content. Samples were hydrolyzed at 110°C for 18 h in 38% HCl and assayed using a chloramine-T assay with a hydroxyproline: collagen ratio of 1:7.69 (Kafienah and Sims, 2004; Ignat’eva et al., 2007). Total sGAG and collagen values and values normalized to DNA content are reported.

### RNA Isolation and Real-time Reverse Transcriptase Polymerase Chain Reaction

Quantitative real-time reverse transcription-polymerase chain reaction (qRT-PCR) was used to determine the relative gene expression changes in RNA isolated from MSCs cultured in tensile strain bioreactors. The central sections of dog-bone shape samples were mechanically disrupted in buffer RLT (Qiagen) supplemented with β-mercaptoethanol (10 µL/mL), frozen in liquid nitrogen, and stored at −80°C before analysis. Thawed samples were then transferred to spin columns for homogenization (QIAShredder, Qiagen), followed by RNA isolation (RNeasy Micro Kit, Qiagen) according to the manufacturer’s instructions, and subsequently resuspended in RNase-free water.

RNA concentrations were quantified using a NanoDropTM (ND-1000) spectrophotometer, and RNA was reverse transcribed into cDNA using the High Capacity cDNA Reverse Transcription Kit (Applied Biosystems, Paisley, UK). Real-time PCR reactions were performed in 20 µL volumes containing 10 µL SYBR Green Master Mix (Applied Biosystems), 0.4 µM forward primer and 0.4 µM reverse primer (KiCqStart SYBR Green, Sigma-Aldrich), RNase-free water, and sufficient sample for 50 ng of cDNA. Primers used and gene abbreviations are listed in Table 1. Reactions were carried out in duplicate on an ABI 7500 real-time PCR system (Applied Biosystems) with an amplification profile of 50°C for 2 min, 95°C for 10 min, followed by 40–45 cycles of denaturation at 95°C for 15 s and annealing/amplification at 60°C for 1 min. Quantitative expression of target genes relative to the endogenous control reference gene (*GAPDH*) and the selected calibrator was carried out using the 2^−ΔΔCT^ method as previously described (Livak and Schmittgen, 2001).

**Table 1:** KiCqStart SYBR green primers used for real-time reverse transcription polymerase chain reactions.

Symbol	Gene	Forward primer	Reverse primer
*ACAN*	Aggrecan	GACCACTTTACTCTTGGTG	TCAGGCTCAGAAACTTCTAC
*ACTA2*	Smooth muscle aortic alpha-actin	CAAAAGAGGAATCCTGACC	CATTGTAGAAAGAGTGGTGC
*ALPL*	Alkaline phosphatase	TTTCACTCTTCTTAGTGCTG	CGTTACGGAATGAGGAAAC
*BMP2*	Bone morphogenic protein-2	ATGTGGAGGCTCTTTCAATG	CATGGTCGACCTTTAGGAG
*CNN1*	Calponin 1, basic, smooth muscle	AGATGGCATCATTCTTTGC	ATGAAGTTGCCAATGTTCTC
*COL10A1*	Collagen type 10, alpha-1	GTAGGTGTTTGGTATTGCTC	GAGCAATACCAAACACCTAC
*COL1A1*	Collagen type 1, alpha-1	TAGACATGTTCAGCTTTGTG	GTGGGATGTCTTCTTCTTG
*COL2A1*	Collagen type 2, alpha-1	CGACGACATAATCTGTGAAG	TCCTTTGGGTCCTACAATATC
*COL3A1*	Collagen type 3, alpha-1	TCATCCCACTGTTATTTTGG	CTCTATCCGCATAGGACTG
*FABP4*	Fatty acid binding protein-4	CTGAAGAGAGTCATTGCAC	CATTTTGTGAGCACTCTAGG
*GAPDH*	Glyceraldehyde 3-phosphate dehydrogenase	TTTAACTCTGGCAAAGTGG	GAACATGTAGACCATGTAGTG
*LECT1*	Leukocyte cell derived chemotaxin-1	ACCTTTAAAATGGGAAACGG	GCTTTGATGTAGCACTTCTC
*LPL*	Lipoprotein lipase	ACCTAACTTCGAGTATGCAG	GGTGAATGTGTGTAAGACG
*MYH1*	Myosin, heavy chain 1	GAGTCACTTTCCAGCTAAAG	CATTTCAATGAGCTCTGGC
*OPN*	Secreted phosphoprotein 1 (osteopontin)	CTGCAGACCAAGGAAAATC	AGCATCTGTGTATTTGTTGG
*RUNX2*	Runt-related transcription factor-2	CCAACAGAGGCATTTAAGG	CCAAAAGAAGTTTTGCTGAC
*SOX9*	SRY (sex determining region Y)-Box-9	CAGACCTTGAGGAGACTTAG	GTTCGAGTTGCCTTTAGTG
*TNC*	Tenascin-C	ATCTAGTCTTTCTCAACTCCG	GAGTAGAATCCAAACCAGTTG

### Fluorescent Staining and Quantification of Cellular Anisotropy of MSC Filamentous Actin

Fluorescent images were taken from the central (strained) section only. F-actin cytoskeletal filaments were visualized using rhodamine 110 conjugated phalloidin (VWR). Dog-boned shaped MSC seeded constructs were sectioned for imaging through the depth of the sample and fixed in 4% paraformaldehyde solution. Cells were permeabilized in 0.5% Triton-X100 (Sigma-Aldrich), then incubated overnight in phosphate-buffered saline (PBS) with 1.5% BSA and rhodamine 110 conjugated phalloidin (1:40; 200 U/mL; VWR). Construct slices were washed in PBS and imaged with an Olympus FluoView FV1000 Confocal Microscope. Anisotropy of actin filaments was quantified by performing analysis of confocal images (*n* = 4) using the ImageJ FibrilTool plugin (Boudaoud et al., 2014).

### Statistical Analysis

All statistical analyses were performed using GraphPad Prism (Version 6.01) software. Data were checked for normal distribution (using D’Agostino–Pearson omnibus *K*^2^ method) before performing parametric tests. Study groups consisted of constructs created using MSCs derived from a single porcine donor, and *n* indicates the number of experimental replicates within each group. Student’s *t*-tests were used where appropriate and one-way analyses of variance with Tukey’s post-test were used to compare multiple conditions. Numerical and graphical results are reported in the form of mean ± SE from the mean. Significance was accepted at a level of *p* ≤ 0.05, and multiplicity adjusted *p*-values thresholds are indicated for individual comparisons. Gene expression data are present as fold differences normalized to the mean of the free-swelling (non-loaded) control group and fold increase or fold decrease, where relevant, are indicated in the text below by ↑ or ↓, respectively.

## Results

### CTS in the Absence of Soluble Differentiation Factors Promotes the Expression of Tenogenic (TNC) and Osteogenic Markers while Inhibiting Adipogenesis

**Figure 2:**
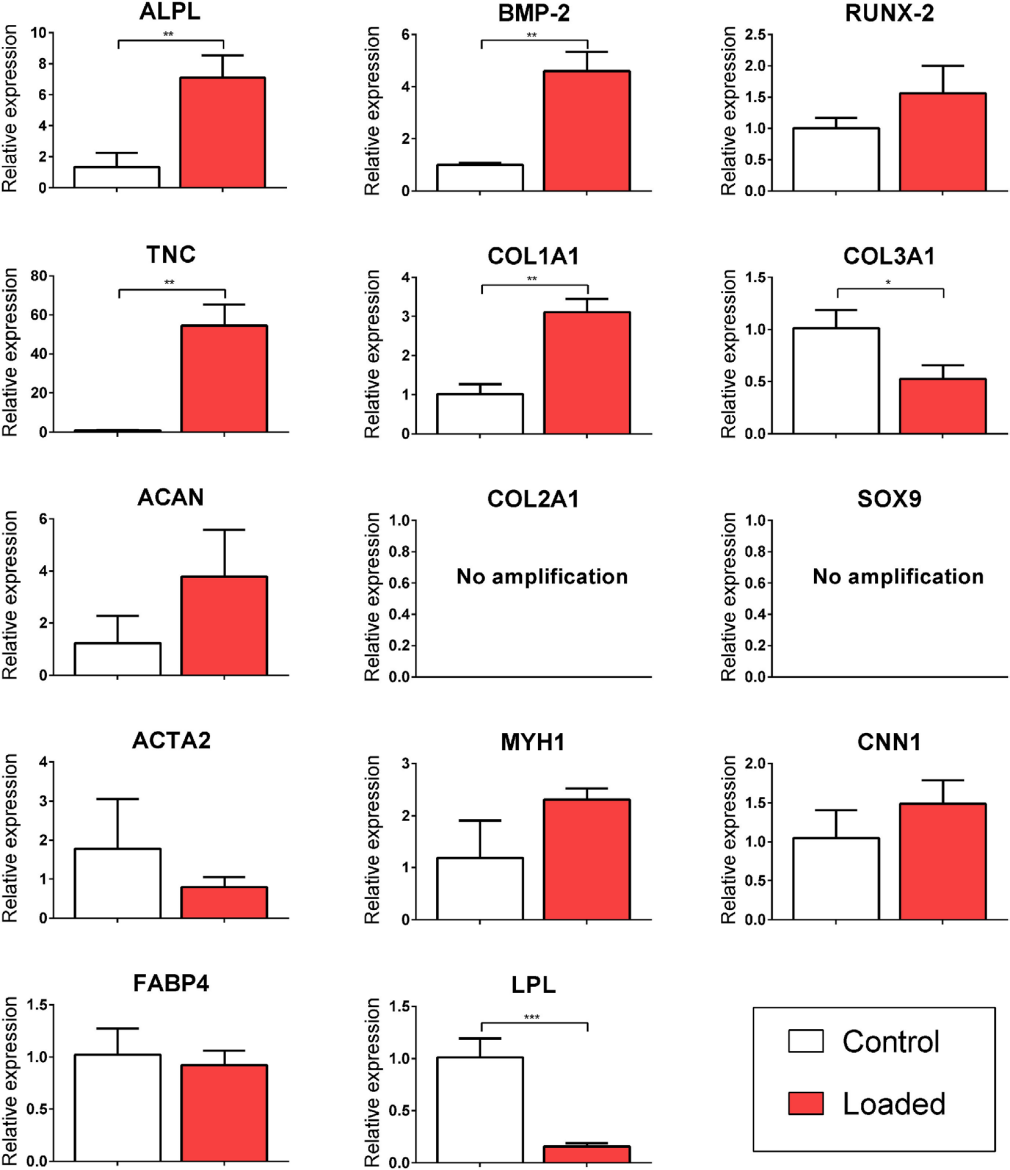
Relative gene expression of multipotent stromal cells (MSCs) encapsulated in fibrin hydrogels and cultured in tensile strain bioreactor in the absence of growth factors for 7 days. The application of 10% cyclic tensile strain at 0.5 Hz was found to promote intramembranous ossification and inhibit adipogenic differentiation of MSCs. Data are presented as fold changes, relative to the non-loaded (control) group. Significant differences are indicated where present (*p < 0.05; **p < 0.01; ***p < 0.001; *n* = 5). See Table 1 for details of gene symbols.

Multipotent stromal cells encapsulated in fibrin hydrogels were cultured in bioreactors in the absence of specific growth factors for 7 days. The application of 10% CTS at 0.5 Hz was found to upregulate the TNC markers *TNC* and *COL1A1* (↑ 54.56 ± 10.83 and ↑ 3.11 ± 0.34, respectively; *p* < 0.003) and to suppress the expression of the adipogenic marker *LPL* (↓ 6.84 ± 0.62, *p* = 0.0001). Mechanical stimulation had no effect on the expression of the myogenic markers *ACTA2, CNN1*, and *MYH1* (Figure 2). CTS was also found to enhance the expression of a number of markers of osteogenesis, including *ALPL* and *BMP2* (↑ 7.09 ± 1.46 and ↑ 4.61 ± 0.74; *p* < 0.003). Furthermore, *COL10A1* was found to be expressed in all loaded samples but was not detected in samples in the non-loaded control group (data not shown). Since no expression of chondrogenic markers *COL2A1* or *SOX9* was detected, while the osteogenic markers *ALPL* and *BMP2* were upregulated following mechanical loading, it would appear that CTS may have a positive role in promoting (intramembranous) osteogenesis of MSCs in the absence of exogenously supplied growth factors. However, since long bones are known to develop and heal *via* the process of endochondral ossification, we therefore next sought to explore the role of CTS on MSCs maintained in chondro-inductive medium for a long-term culture duration.

### CTS Promotes Endochondral Ossification of Chondrogenically Primed MSCs in a Magnitude-Dependent Manner

**Figure 3:**
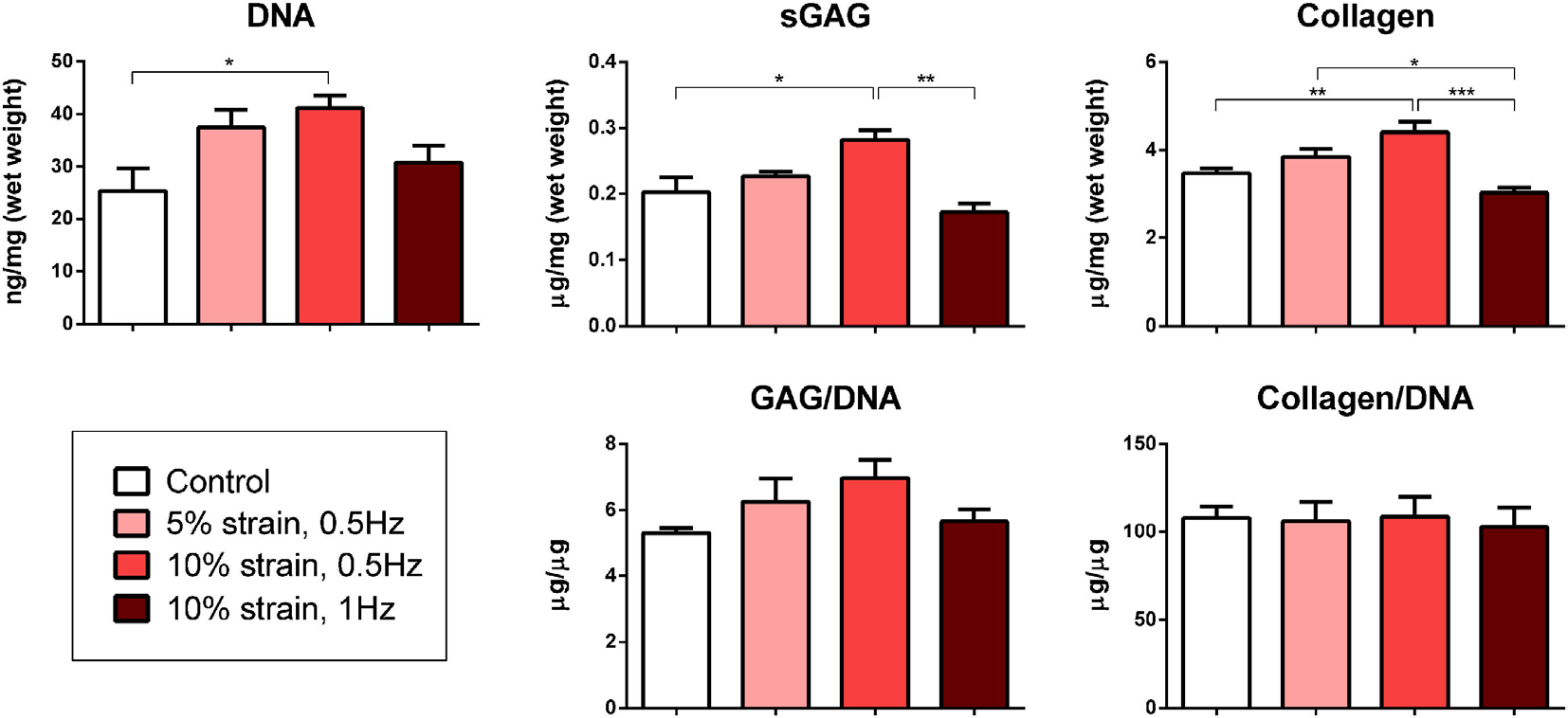
Biochemical analysis of multipotent stromal cell encapsulated fibrin hydrogels cultured in tensile strain bioreactors in media supplemented with chondrogenic growth factor transforming growth factor-β3 for 21 days. The application of 10% cyclic tensile strain at 0.5 Hz was found to promote cellular proliferation, which led to an increase in total cartilage matrix deposition. Significant differences are indicated where present (*p < 0.05; **p < 0.01; ***p < 0.001; *n* = 5).

**Figure 4:**
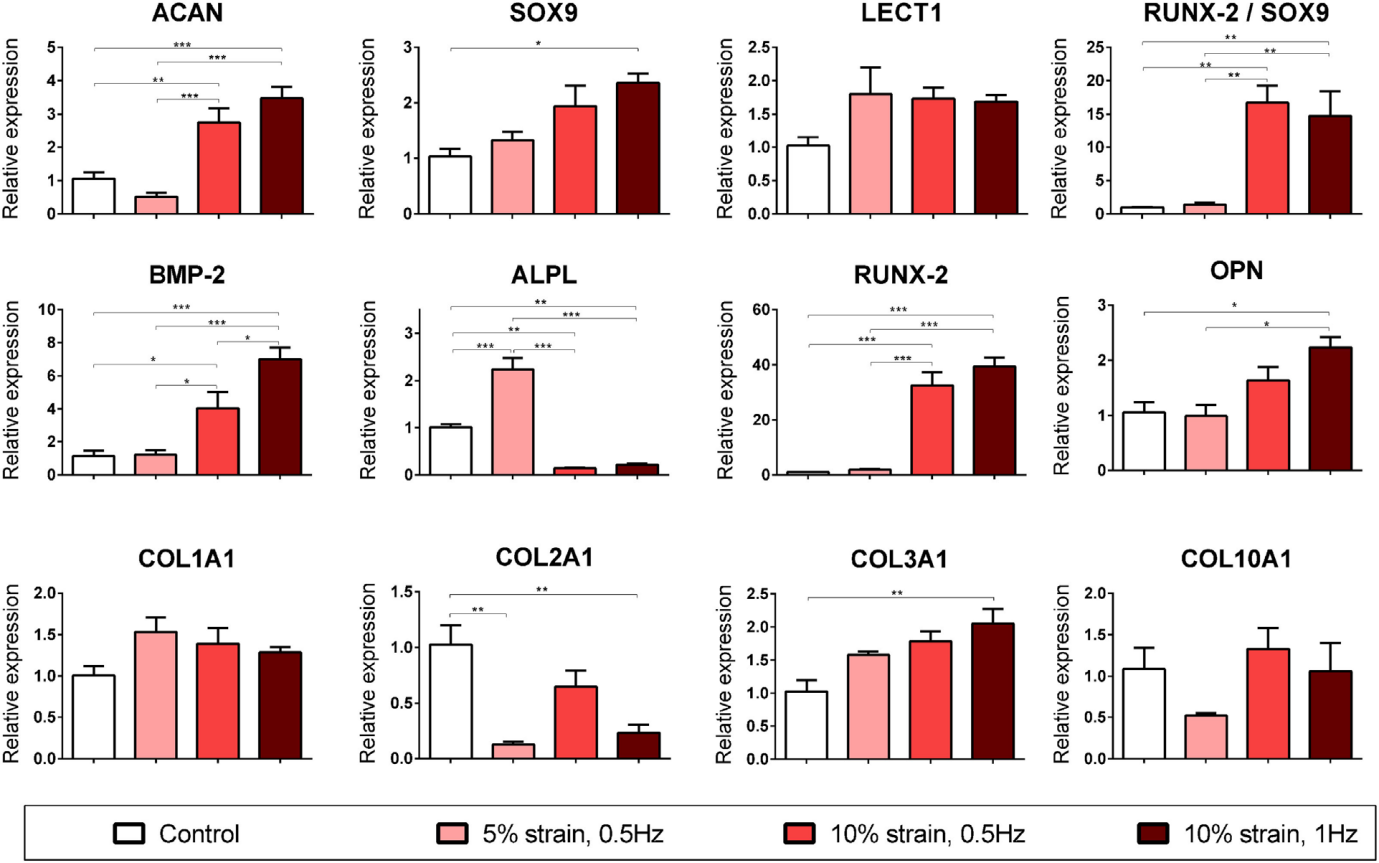
Chondrogenic and osteogenic gene expression of multipotent stromal cells (MSCs) encapsulated in fibrin hydrogels and cultured in media supplemented with chondrogenic growth factor transforming growth factor-β3 for 21 days. Cyclic tensile strain was found to promote endochondral ossification of chondrogenically primed MSCs in a magnitude- and frequency-dependent manner. Data are presented as fold changes, relative to the non-loaded (control) group. *RUNX2/SOX9* ratio is presented as fold change/fold change. Significant differences are indicated where present (*p < 0.05; **p < 0.01; ***p < 0.001; *n* = 5). See Table 1 for explanation of gene symbols.

Multipotent stromal cells encapsulated within fibrin hydrogels and maintained in media supplemented with the chondrogenic growth factor TGF-β3 for 21 days were found to synthesize cartilage extracellular matrix components (Figure 3) and express genes associated with a chondrogenic phenotype (Figure 4). The application of 10% strain at 0.5 Hz was found to promote cellular proliferation, which led to increased overall GAG and collagen synthesis (Figure 3). However, when normalized to DNA content, there was no difference in ECM synthesis rates on a per cell basis.

10% CTS at 1 Hz was also found to significantly enhance several markers of chondrogenesis (*ACAN* and *SOX9*) but inhibit *COL2A1* expression in MSCs cultured in chondrogenic media for 3 weeks (Figure 4). Furthermore, CTS was found to enhance the expression of markers associated with an osteogenic phenotype in a magnitude- and frequency-dependent manner (Figure 4). Application of smaller strains appeared to be insufficient to invoke a response in many of the osteogenic markers investigated, with only *ALPL* upregulated following the application of 5% CTS (↑ 2.24 ± 0.18, *p* < 0.0001). Of the loading regimens investigated, a tensile stimulus of 10% strain at 1 Hz was observed to invoke the largest increase in osteogenic gene expression and resulted in a significant upregulation of *BMP2, RUNX2, COL3A1*, and *OPN* (↑ 7.02 ± 0.92, ↑ 39.41 ± 0.4.34, ↑ 2.05 ± 0.25, and ↑ 2.24 ± 0.31; *p* < 0.015) when compared with the non-loaded control group. Furthermore, the ratio of *RUNX2* expression to *SOX9* expression was dramatically increased in the 10% CTS groups. The magnitude of strain appeared to effect gene expression to a greater degree than frequency, with the only apparent difference due to strain frequency being a smaller increase in *BMP2* expression in samples strained at 0.5 Hz compared with 1 Hz. However, *BMP2* levels in this 10% CTS group still remained greater than those in hydrogels strained by both 0 and 5%. In contrast to the 5% CTS group, the application of 10% strain lead to a significant downregulation in *ALPL* expression after 21 days compared with non-loaded controls.

### CTS Promotes Cellular Alignment of MSCs in 3D Fibrin Hydrogels

**Figure 5:**
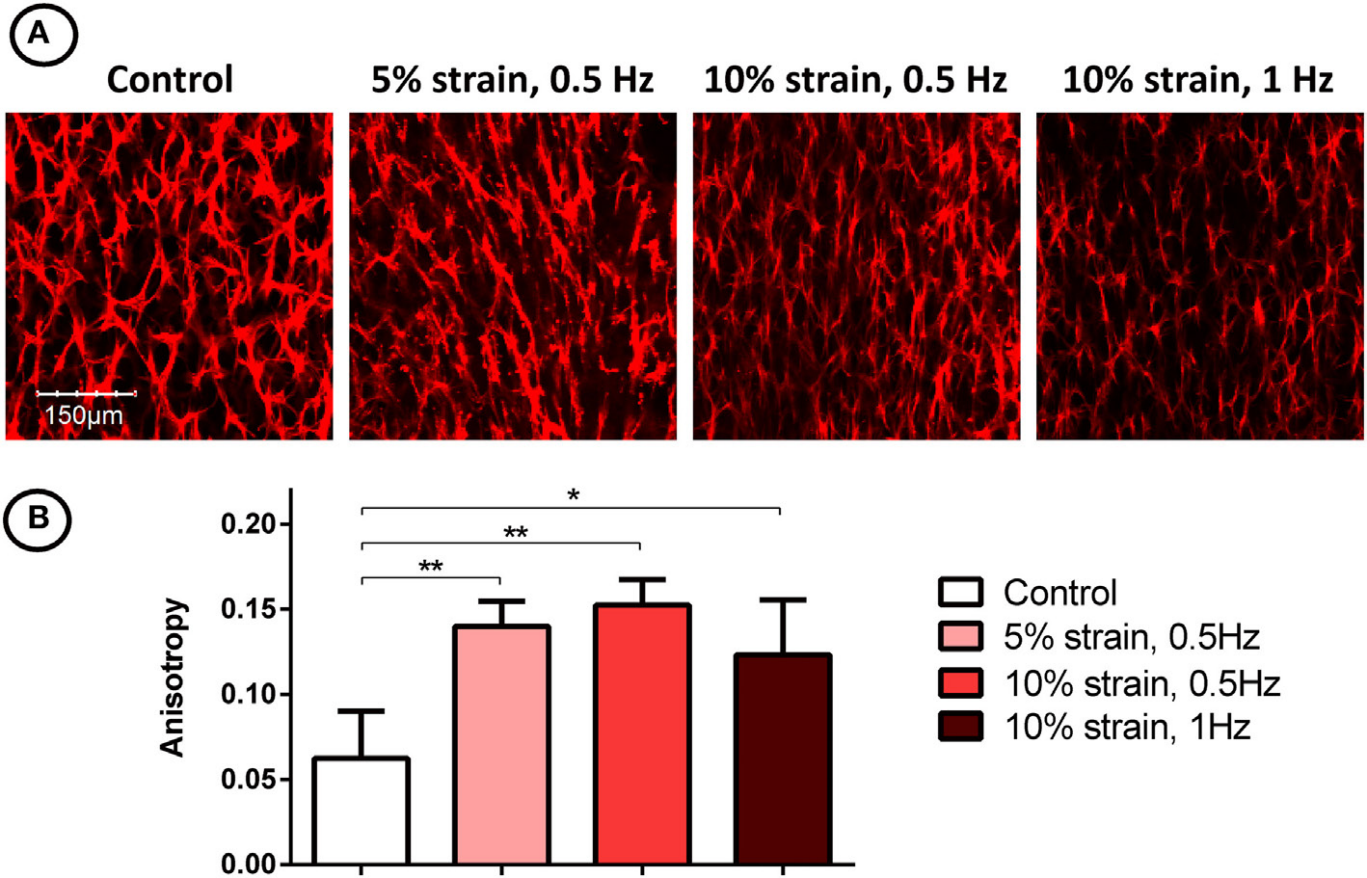
F-actin staining **(A)** and quantification of filament anisotropy **(B)** of multipotent stromal cells encapsulated in fibrin hydrogels and cultured in tensile strain bioreactors in media supplemented with chondrogenic growth factor transforming growth factor-β3 for 21 days. The application of cyclic tensile strain was found to increase cellular organization. Significant differences are indicated where present (*p < 0.05; **p < 0.01; *n* = 5).

Filamentous-actin staining and confocal imaging of MSC laden 3D hydrogels showed spindle-shaped, spread cells with well-developed actin networks in all groups (Figure 5A). Image analysis and quantification of cellular organization revealed that the application of CTS resulted in increased cellular anisotropy/alignment in all groups subjected to mechanical strain (Figure 5B).

## Discussion

Our long bones develop and heal through the process of endochondral ossification. The objective of this study was to explore the role of mechanical cues in regulating the initiation and progression of endochondral ossification. Our hypothesis was that uniaxial CTS would promote an endochondral phenotype in MSCs encapsulated within physiologically relevant 3D fibrin hydrogels. While it has previously been reported that CTS promotes a fibro-chondrogenic phenotype in MSCs seeded in 3D hydrogels (Connelly et al., 2010), the role of this mechanical stimulus in regulating the conversion of cartilage into bone remains poorly understood. The findings of this study suggest that CTS can promote osteogenesis of MSCs, either directly or indirectly through a cartilage intermediate, and hence plays a key role in both intramembranous and endochondral ossification of MSCs.

The application of CTS to MSCs cultured in the absence of soluble differentiation factors was found to increase the expression of osteogenic and TNC markers. Furthermore, in agreement with recently reported findings in 2D culture systems (Li et al., 2015), it was found that tensile loading suppressed adipogenesis of MSCs. This suggests that CTS is a positive stimulus for intramembranous ossification of MSCs, although not uniquely promoting differentiation down this pathway. Enhanced osteogenesis and a suppression of adipogenesis have also been observed in response to other mechanical cues such as increases in substrate stiffness (Huebsch et al., 2010; Dupont et al., 2011). Increased osteogenesis on stiff substrates has been linked to cytoskeletal tension and RhoA and Rho-associated protein kinase (ROCK) signaling, a pathway that is known to regulate remodeling of the actin cytoskeleton and play a key role as a dynamic mechanosensor (Peyton et al., 2007; Guilak et al., 2009; Khatiwala et al., 2009; Holle and Engler, 2011). Mechanical cues such as cyclic stretch and fluid flow have previously been shown to promote cell stiffening and enhance cytoskeletal tension in a RhoA/ROCK-dependent manner, suggesting that common molecular mechanisms translate diverse mechanical cues into increases in osteogenesis (Chiquet et al., 2004; Lee et al., 2006; Matthews et al., 2006; Arnsdorf et al., 2009). These findings add to the growing body of work pointing to the role of different mechanical cues, from substrate stiffness (Huebsch et al., 2010; Park et al., 2011) to CTS, in directing either an osteogenic or adipogenic phenotype in MSCs.

Given that long bones form and generally regenerate *via* endochondral ossification, we examined the influence of long-term CTS on chondrogenically primed MSC laden 3D hydrogels to explore how this mechanical cue regulates the process of endochondral ossification. While CTS did not appear to initiate chondrogenesis in the absence of soluble differentiation factors, in the presence of exogenously supplied TGF-β3 this mechanical cue was found to influence the expression of both chondrogenic and osteogenic genes in a strain magnitude- and frequency-dependent manner. 10% tensile strain at 1 Hz was found to promote both chondrogenic (*ACAN* and *SOX9*) and osteogenic (*BMP2, RUNX2, OPN*, and *COL3A1*) gene expression, whereas 5% strain was determined to be insufficient to induce upregulation of many of these markers. Interestingly, mechanical stimulation was observed to lead to an inhibition of *COL2A1* expression. *COL2A1* expression has previously been shown to follow a temporal profile during endochondral ossification, with peak expression observed at day 7 after *BMP2* induced bone formation (Clancy et al., 2003). MSCs in hydrogels strained at 10% also exhibited a downregulation in *ALPL* expression after 21 days, whereas those strained at 5% showed enhanced *ALPL* expression (Figure 4). *ALPL* is considered to be an early marker of osteogenesis in MSCs and is upregulated during the differentiation phase and then downregulated before mineralization (Kulterer et al., 2007). Therefore, the downregulation in *ALPL* expression observed in MSCs subjected to higher magnitude strains may further suggest that CTS is accelerating the process of endochondral ossification. Furthermore, the ratio of *RUNX2* to *SOX9* was dramatically increased (>14-fold increase) following application of 10% CTS. *SOX9* has been shown to be highly expressed in pre-hypertrophic chondrocytes, but not in hypertrophic chondrocytes (Zhao et al., 1997; Hattori et al., 2010). It exerts an inhibitory effect on *RUNX2* function (Zhou et al., 2006) and therefore has been reported to be a negative regulator of bone marrow formation and endochondral ossification (Hattori et al., 2010). There is also evidence to suggest that increased expression of *RUNX2* combined with *SOX9* downregulation in hypertrophic chondrocytes is a prerequisite to initiation of the cartilage–bone transition in the growth plate (Hattori et al., 2010). Furthermore, the *RUNX2*:*SOX9* ratio has been reported to be a positive indicator of osteogenic potential in MSCs (Loebel et al., 2014).

The application of uniaxial CTS was also found to promote re-organization of actin filaments and increase overall anisotropy in the actin network, consistent with previously reported studies of MSCs cultured on both 2D substrates (Hamilton et al., 2004; Chen et al., 2008) and in 3D environments (Altman et al., 2002). Anisotropic cellular and matrix organization is a characteristic of many developing biological tissues. For example, it has been shown that collagen orientation in the perichondrium of long bones align with the direction of growth, and it has been suggested that this is due to the combination of mechanical tissue strain and the synthesis of new tissue matrix (Foolen et al., 2008). Growth plate tissue is also highly anisotropic, with distinct cellular organization; chondrocytes are arranged into vertical columns, which act as functional units of longitudinal bone growth (Hunziker, 1994). Since chondrocyte alignment is a hallmark of hypertrophy and endochondral ossification in developing limbs, it is likely that novel cell culture systems that direct cellular anisotropy and guide structural alignment will be required when engineering grafts to repair structurally complex tissues.

In either the presence or absence of exogenously supplied TGF-β3, increases in the expression of osteogenic markers in response to mechanical loading correlated with increases in *BMP2* expression. CTS induced increases in osteogenic growth factor expression is a likely mechanism that warrants further investigation. Other loading modalities such as dynamic compression and shear loading have also been shown to lead to increased expression of other members of the TGF-β superfamily in MSCs (Huang et al., 2004; Li et al., 2009, 2010). The molecular mechanisms by which mechanical loading leads to increases in growth factor expression, and whether different loading modalities such as cyclic tension and compression lead to distinct growth factor expression profiles, are important questions that warrant further study.

The novel bioreactor system developed as part of this study has a number of potential applications, both as a tool for fundamental mechanobiology experiments and in the field of tissue engineering. The majority of studies reported in the literature that explore the role of CTS in regulating cellular behavior involve the application of stretch to 2D substrates (Simmons et al., 2003; Friedl et al., 2007; Qi et al., 2008; Diederichs et al., 2009), whereas the system described in this study involves encapsulating cells in more physiologically relevant 3D hydrogels. The bioreactor system should be compatible with any hydrogel used for cell encapsulation, thereby enabling studies exploring how CTS and factors such as matrix stiffness (Huebsch et al., 2010) or ligand presentation (Bian et al., 2013) might interact to regulate stem cell fate. In this study, we selected fibrin hydrogels as our substrate as they mimic the clot that forms within a fracture callus and hence represent a useful model when exploring the role of environmental cues in regulating bone healing. When used in conjunction with an oxygen tension controllable incubator, the fact that the bioreactor supplies filtered air recycled from within the incubator enables studies exploring the interaction between CTS and altered levels of oxygen availability, another key regulator of intramembranous and endochondral ossification (Meyer et al., 2010; Sheehy et al., 2012). For all of these reasons, the bioreactor should find broad utility in musculoskeletal tissue engineering; not only for producing grafts for endochondral bone tissue engineering (Scotti et al., 2010; Farrell et al., 2011; Thompson et al., 2015, 2016; Cunniffe et al., 2017) but also for generating other biological tissues that are subjected to large tensile loads, such as ligament and tendon.

## Conclusion

While it is clear that the application of extrinsic mechanical loading can guide stem cell fate, a more complete understanding of how MSCs respond to specific mechanical signals is needed for successful cell based strategies in musculoskeletal medicine. The data presented here demonstrates that, depending on the context in which the stimulus is applied, cyclic tensile strain can be a positive stimulus for the promotion of both intramembranous and endochondral ossification of MSC. These new insights can inform the development of novel tissue-engineering strategies for cartilage and bone regeneration.

## Author Contributions

SC—developed bioreactor, performed experiments and data analysis, author. CB—co-supervisor, coauthor. DK—co-supervisor, coauthor, principal investigator, and corresponding author.

## Conflict of Interest Statement

The authors declare that the research was conducted in the absence of any commercial or financial relationships that could be construed as a potential conflict of interest.
